# Artificial Nesting Hills Promote Wild Bees in Agricultural Landscapes

**DOI:** 10.3390/insects13080726

**Published:** 2022-08-14

**Authors:** Ulrich Neumüller, Hannah Burger, Antonia V. Mayr, Sebastian Hopfenmüller, Sabrina Krausch, Nadine Herwig, Ronald Burger, Olaf Diestelhorst, Katrin Emmerich, Mare Haider, Manuel Kiefer, Jonas Konicek, Johann-Christoph Kornmilch, Marina Moser, Christoph Saure, Arno Schanowski, Erwin Scheuchl, Julia Sing, Max Wagner, Julia Witter, Hans R. Schwenninger, Manfred Ayasse

**Affiliations:** 1 Institute of Evolutionary Ecology and Conservation Genomics, Ulm University, 89081 Ulm, Germany; 2 Institute for Ecological Chemistry, Plant Analysis and Stored Product Protection, Julius Kühn-Institute, 14195 Berlin, Germany; 3 Independent Researcher, IFAUN-Faunistik und Funktionale Artenvielfalt, 67246 Dirmstein, Germany; 4 Independent Researcher, 40591 Düsseldorf, Germany; 5 Independent Researcher, 64285 Darmstadt, Germany; 6 Independent Researcher, Institut für Landschaftsökologie und Naturschutz Bühl, 77815 Bühl, Germany; 7 Independent Researcher, 17489 Greifswald, Germany; 8 Independent Researcher, Büro für Tierökologische Studien, 12167 Berlin, Germany; 9 Independent Researcher, 84030 Ergolding, Germany; 10 Independent Researcher, Kompetenzzentrum Wildbienen gGmbH, 67433 Neustadt an der Weinstraße, Germany

**Keywords:** ground-nesting, native bees, conservation, nesting aid, soil, construction guide

## Abstract

**Simple Summary:**

The majority of wild bees nest underground in the soil. Many of these species are threatened by loss of habitat. Adequate nesting sites are particularly limited in agricultural landscapes. We therefore established 20 artificial nesting hills in Germany to promote ground-nesting bees. A detailed construction guide for the hills is presented. On the basis of our study results, we recommend the establishment of hills on sun-exposed sites and to ensure their existence for many years. We constructed hills from local soil with dimensions of 9 × 3 × 1.6 m. During a two-year bee monitoring initiative, we recorded a high diversity of soil-nesting bee species. We conclude that artificial nesting hills can act as a valuable nesting resource for wild bees.

**Abstract:**

The availability of nesting resources influences the persistence and survival of bee communities. Although a positive effect of artificial nesting structures has frequently been shown for aboveground cavity-nesting wild bees, studies on below ground-nesting bees are rare. Artificial nesting hills designed to provide nesting habitats for ground-nesting bees were therefore established within the BienABest project in 20 regions across Germany. Wild bee communities were monitored for two consecutive years, accompanied by recordings of landscape and abiotic nest site variables. Bee activity and species richness increased from the first to the second year after establishment; this was particularly pronounced in landscapes with a low cover of semi-natural habitat. The nesting hills were successively colonized, indicating that they should exist for many years, thereby promoting a species-rich bee community. We recommend the construction of nesting hills on sun-exposed sites with a high thermal gain of the substrate because the bees prefer south-facing sites with high soil temperatures. Although the soil composition of the nesting hills plays a minor role, we suggest using local soil to match the needs of the local bee community. We conclude that artificial nesting structures for ground-nesting bees act as a valuable nesting resource for various bee species, particularly in highly degraded landscapes. We offer a construction and maintenance guide for the successful establishment of nesting hills for bee conservation.

## 1. Introduction

A species-rich wild bee community depend on diverse flower and nesting resources [1]. However, suitable habitats for bees are decreasing in agricultural landscapes, and many bee species are currently under threat [2,3]. This has consequences for ecosystem stability and food security because bees are essential pollinators [4]. To provide supplementary floral resources for bees, flower strips are established with the expenditure of great effort [5,6,7,8,9]. Besides floral nutrition, the availability of nesting resources influences the persistence and survival of bee populations [10,11,12,13,14]. Bees are often even more limited by the absence of their habitat or nesting site than by that of their host plants [10]. Hence, the provision of artificial nesting structures has potential for the promotion of bees in degraded landscapes [14,15].

About 75% of all bee species in Central Europe use soil nests. Globally estimated, 83% of nonparasitic bees are belowground nesters, whereas 91% of parasitic bees choose such nests [13,16]. Most ground-nesting bees dig subterranean tunnels that are connected to brood chambers, which they provision with pollen and nectar [13,17]. Parasitic bees invade the nests of belowground nesting bees, and their offspring use the larval provision of their host bees.

Artificial nesting structures are usually offered for aboveground-cavity-nesting wild bees and thus have been investigated in many studies, whereas artificial nesting structures for belowground nesting bees have rarely been examined and only in small-scale studies [15,18]. Hence, significant knowledge gaps exist with regard to the establishment of artificial nesting resources to house a high diversity and abundance of wild bees [19].

Various abiotic factors influence the nesting activities of wild bees [13]. For example, soil texture affects the temperature, humidity, and oxygen availability in the substrate, all of which can influence the survival of offspring in the nest [13]. Although bees are often associated with sandy and loamy soils, the different bee species show various levels of specialization for nesting resources, and highly interspecific variations in the soil textures of nesting sites have been observed [13,17,20]. Whereas some species accept a variety of substrates [17], others show strong preferences [1,16,21]; the preferred substrate is nevertheless unknown for most species [16]. In an experimental study, the number of bee species that colonized artificial soil squares seemed not to be influenced by the soil texture [15]. However, not only species richness but also bee community composition might vary with soil texture.

Soil temperature, which is driven by air temperature and sun exposure, also influences the nest site selection of bees [13,16]. The temperature of the nesting substrate affects bee size, development rate, and the mortality of the offspring [22]. Warm soil conditions have positive effects on the development of the brood [23], and, at the nest entrance, higher temperatures allow an earlier start of the foraging activity of adult females [24,25]. Nevertheless, temperatures exceeding 40 °C are predicted to result in an abrupt increase in brood mortality [16,22]. Although these findings appear relevant for aboveground cavity-nesting wild bees, it remains unclear as to whether nests of ground-nesting bees can reach such high temperatures in Central Europe [16].

The wild bee community in the surrounding landscape also has an effect on the colonization of nesting structures. Near-natural habitats can sustain large numbers of bee species [6] and can therefore act as a source area from where wild bees can start to colonize new nesting grounds. However, the surrounding landscape must provide sufficient feeding resources to allow the bee communities to thrive within a new nesting structure [26]. Large quantities of floral resources have also been shown to increase the reproductive success of wild bees in artificial trap nests [27]. Hence, the simultaneous provision of artificial nesting structures together with flower planting on arable land appears to be a promising approach to support wild bees in agricultural landscapes.

We explored wild bee communities of nesting hills designed to provide an artificial nesting habitat for ground-nesting bees in 20 regions across Germany. We investigated the way in which the bees colonize the nesting hills. We monitored wild bees on the nesting hills for two consecutive years and recorded various abiotic parameters. To evaluate the colonization, we compared the established bee community of the nesting hills with bee communities of three different habitats in close proximity: existing semi-natural grasslands, residual habitats such as field margins or rarely used field paths, and perennial flower plantings/flower strips established within the project. Moreover, we studied whether the landscape composition influences the colonization of the hills. Furthermore, we investigated whether nesting bees collect pollen from the nearby flower plantings. The study was embedded in the long-term project BienABest (Standardisierte Erfassung von Wildbienen zur Evaluierung des Bestäuberpotenzials in der Agrarlandschaft; www.bienabest.de, accessed on 1 July 2022), which aims to increase wild bee diversity and to secure the ecosystem service of pollination in agricultural landscape.

We hypothesized that (1) bee species richness and abundance on the hills are higher in the second compared to the first year because bees need some time to colonize the newly established structures. As wild bee development and activity is strongly moderated by abiotic parameters, we further hypothesized that (2) an optimum exists with regard to sun exposure and the substrate temperature of a nesting site. Because some bee species show preferences for specific soil types, we assumed that (3) the soil composition influences the bee communities of our nesting hills. Furthermore, we expected that (4) a high cover of semi-natural habitat in the surrounding landscape has a positive effect on the species richness and abundance of bees. In addition, we assumed that (5) the established nearby flower plantings represent an important pollen source for the nesting bees. In addition to testing these hypotheses, we provide technical guidelines for the establishment and management of nesting hills in order to create an attractive nesting resource for wild bees.

## 2. Materials and Methods

### 2.1. Nesting Hills

The study was conducted in 20 different research areas, distributed across seven federal states of Germany during the years 2019 and 2020 (for location of research areas, see Table S1 and [6]). The study area spread over 710 km longitudinally and 510 km latitudinally, with elevations ranging between 20 and 580 m above sea level. One nesting hill was established in each research area in the year before the examinations started.

**Figure 1:**
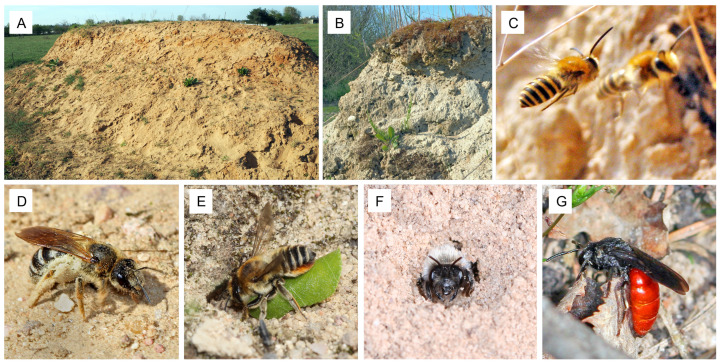
Diverse bee species colonized the artificial nesting hills over time. All hills had the same size (9 m long, 3 m wide, and 1.6 m high) and offered horizontal and vertical nesting sites ((**A**) photo A. Mayr). In particular, vertical slopes ((**B**) photo A. Mayr) were abundantly colonized. Large numbers of bee species with solitary, social, and parasitic life-styles nest underground ((**C**) *Colletes hederae* males (photo R. Burger), (**D**) *Halictus quadricinctus* (photo: J.-C. Kornmilch), (**E**) *Megachile maritima* (photo J.-C. Kornmilch), (**F**) *Andrena vaga* (photo H. Burger), (**G**) *Sphecodes albilabris*, photo H. Burger).

The artificial nesting structures were 9 m long, 3 m wide, and 1.6 m high (Figure 1). Each of the hills was established with soil of the region (maximum transfer distance about 30 km) on a sun-exposed site. We used local soil to match the nesting substrate of the local bee community and established the hills on public sites to ensure their existence for several years even after the end of the project. Detailed construction and management guidelines are given in File S1. All nesting hills were constructed in the close proximity (mean distance of around 400 m) of other research plots of the BienABest project [6] to allow comparisons of the bee communities.

### 2.2. Bee Survey on Nesting Hills

Wild bee data were collected from the nesting hills twice a year, during the early summer between the middle of May and middle of June and during midsummer between the middle of July and middle of August for two consecutive years. Areas of 4 × 1 m were monitored for 25 min each in the centers of both lengths of the hills. All bees were recorded that were observed at the sampling plot entering or leaving nest holes or that were flying around. Bees were sampled with an aerial net and stored in vials in a cooling bag during the sampling event to avoid double counting. After the 25 min observation period, all bees were placed in observation tubes and inspected with a magnifying glass or a field microscope and released after determination. Bees that could not be identified in the field were killed and taken to the laboratory for further identification. Sampling was only conducted during warm temperatures (higher than 12 °C), sunny weather (cloud cover less than 30%), and low wind conditions.

We performed more intensive monitoring with either one or two additional monitoring events per year in five of the research areas to control for seasonal variation (see Table S1 for a detailed list of the sampling scheme). During the extended monitoring approach, nesting hills were sampled twice during each sampling round to account for intra-day variation once before and once after midday.

### 2.3. Abiotic Parameters (Substrate, Soil Temperature, Aspect)

Substrate samples from all 20 nesting hills were taken during the first monitoring year and stored in plastic bags until analysis. An air-dried substrate sieved to 2 mm was used. The substrate samples before screening and the remaining material (pebbles > 2 mm) were weighed to calculate the relative number of pebbles larger than 2 mm in size. The screened substrates were stored at room temperature until they were analyzed in the laboratory to determine the soil pH according to A5.1.1 (VDLUFA 1991). For this purpose, 10 g substrate was weighed into a 50 mL beaker, mixed with 25 mL 0.01 M CaCl_2_ solution, and stirred twice within one hour with a glass rod. The pH value was measured at room temperature (WTW inoLab 720 pH meter). The particle size determination was carried out according to DIN 19683-2 (1997) by wet sieving to 0.063 mm and sedimentation analysis for grain fractions 0.002 < d < 0.02 mm after pre-treatment with sodium pyrophosphate. The determination of the organic substance in humus-rich carbonate-free sandy soils and peat was carried out according to DIN 19684-3 (1998). For the analysis, 10 g substrate was weighed into ceramic crucibles and dried first at 105 °C to constant weight. Subsequently, the samples were ashed at a temperature of 550 °C. The difference in weight before and after drying corresponded to the organic soil substance.

Soil temperature was measured at a central position of the sampling plots at a depth of 5 cm below the surface as a proxy for substrate temperature at nest entrances, and at a depth of 15 cm as a proxy for substrate temperature in the nests for each monitoring event. Additionally, all nesting hills were mapped in QGIS, and the aspect (compass direction) of each sampling plot was measured in degrees from North.

### 2.4. Comparison of Nesting Hill and Local Bee Community

To evaluate whether the established nesting hills were successful in attracting the local ground-nesting bee community of the surrounding landscape, we compared the bee data of the nesting hills with bee data of reference habitats in the close surroundings: perennial flower plantings (flower strips), semi-natural grasslands, or residual habitats. Residual habitats represented habitat types commonly present in agricultural landscapes. They comprised small areas with ruderal vegetation, field margins, and rarely used field paths. Semi-natural grasslands were characterized by a high diversity of flowering plants and extensive management. Flower plantings were established within the BienABest project during autumn 2017 and spring 2018. They were sown with a mixture of regionally native, naturalized, and cultured (non-invasive) plant species. A detailed methodology describing the establishment of the flower plantings is given in [28]. In each of the described habitat types, three sampling plots of 0.3 ha were established, resulting in a total of 180 reference plots. These plots had a mean distance of around 400 m to the nesting hills.

The bee monitoring of the reference habitat plots was conducted within the same time frame as the nesting-hill monitoring (on the same day or a few days before or after). Variable transect walks were performed for 25 min in the morning and repeated in the afternoon, following the distribution of potential feeding and nesting resources for bees [6,29]. All encountered bees (at flowers, nesting sites, in flight, etc.) were recorded. Each plot was sampled in two subunits of 25 min, one before and one after midday, to account for any intra-day phenologies of bees. Bee data from 2019 were taken from [28].

### 2.5. Landscape Composition (Cover of Semi-Natural Landscape Elements)

The landscape composition was mapped in a 500 m radius around the sampling plots (for a detailed description and mapping procedure, see [6]). The following landscape elements were defined as semi-natural: extensively managed grassland, ruderal areas, woodland edges, and field margins.

### 2.6. Pollen Survey

Pollen samples from the bees were taken during the extended monitoring approach, four times a year. The pollen was carefully removed from the corbiculae of bees that were caught during the sampling events. In cases in which the bees removed the pollen by themselves, the pollen was taken from the glass vials in which the bees were stored. To facilitate the identification of the pollen collected by the bees, reference samples were collected from blooming flowers in about 100 m radius around the nesting hills. The pollen samples were transferred to 1.5 mL Eppendorf tubes and stored at −20 °C. For identification, the collected pollen was mounted on microscope slides by means of fuchsin jelly cubes and examined under a microscope [30].

### 2.7. Statistical Analysis

Only bee species that dug subterranean burrows and the parasitic bee species depending on them were considered for the following analyses. Individuals of *Bombus* spp. were not considered because the observed individuals did not nest on the hills. All statistics were carried out with R [31].

#### 2.7.1. Comparison of the Nesting Hill and the Local Bee Community

We calculated randomized individual-based accumulation curves using the package iNEXT [32] by aggregating all wild bee data (ground-nesting species) for each habitat type in order to compare the species richness between the nesting hills and the reference habitats.

In a separate analysis, we tested whether the bee species composition of the nesting hills was similar to one of the three reference habitats. For this purpose, we performed a pairwise PERMANOVA [33] on the standardized data matrix. Bee data were transformed using the Hellinger transformation [34,35].

#### 2.7.2. Abiotic Parameters (Substrate, Soil Temperature, Aspect), Colonization Time, and Landscape Composition (Cover of Semi-Natural Landscape Elements)

In order to model our data, we calculated generalized additive models (GAMs) implemented in the package mgcv [36]. We set either species richness or abundance as dependent variables, and soil temperature; the five substrate variables of soil pH; the percentage of clay, sand, silt, and pebbles larger than 2 mm; organic matter; and the aspect of the plot (degrees from North) as independent variables. The variables were added to the model by using thin plate splines, except for the aspect, a cyclically variable, which was added as a cyclic cubic regression spline so that the ends of the cyclic spline were joined up.

In the first step, we checked all variables for collinearity by inspecting a heatmap based on Pearson correlation coefficients. Correlation coefficients between variables of |r| > 0.7 were regarded to be problematic for the model estimation [37]. The percentage of sand showed a strong negative correlation with that of silt (r = −0.86) and was therefore excluded from subsequent analyses. Soil temperature at a 5 cm and 15 cm depth also showed a high correlation (r = 0.8). In this case, we decided to perform the model selection process twice. Subsequently, we chose the resulting model that explained a higher proportion of the total deviance in the bee data. The explained deviance for the models including soil temperature at a 5 cm depth was marginally higher than the explained deviance for those ones at a 15 cm depth (model testing species richness: +2.4%, model testing abundance: +0.8%). Hence, we included soil temperature at a 5 cm depth in our final models.

To explore whether the landscape composition had an effect on the colonization of the nesting hills, we included the cover of semi-natural landscape elements with an interaction of sampling year to the model. Sampling year was added additionally as a random effect. We also added the plot ID as a random effect to account for pseudo replication. Furthermore, we added the day of the year of each sampling event as a thin plate spline to the model structure to account for different sampling dates. Subsequently, we applied a backwards AIC model selection to exclude non-informative variables. On the basis of the model selection, all tested substrate parameters for bee species and individuals and the interaction of semi-natural landscape elements with sampling year for bee species were excluded from the modeling process. For all smooth variables, we let the gam function select the number of basis functions (k). We used the function gam.check implemented in the package mgcv [36] to ensure an adequate fit of the models. No adaptations to the models were necessary.

To study the influence of soil composition on bee species composition, we performed a partial redundancy analysis implemented in the vegan package [35], in which we tested the standardized data matrix against the substrate parameters. Bee data were transformed using a Hellinger transformation [34,35]. We added the coordinates of each nesting hill as a condition argument to exclude spatial effects in our data [35]. Subsequently, we used the ordistep function to perform a stepwise AIC model selection of the substrate variables.

## 3. Results

**Figure 2:**
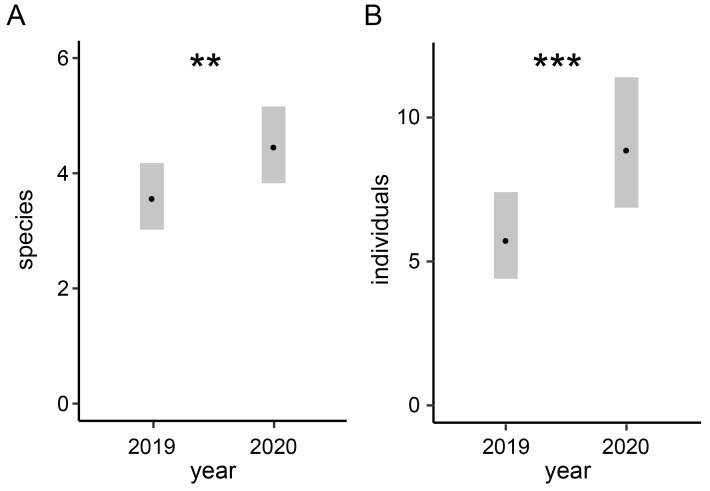
Comparison of (**A**) species richness and (**B**) abundance of bees registered on the nesting hills between the two years. Predicted values with 95% confidence intervals. Bars with asterisk indicate significance: **: *p* < 0.01, ***: *p* < 0.001. Coefficients of the underlying post hoc analysis based on the GAM models are given in the Supplementary Material (Table S3).

We recorded a total of 1959 ground-nesting bees belonging to 119 species, thereof 37 Red-List species (categorized according to [38]), across all 20 nesting hills and over two years. The three most common belowground-nesting bee species were *Halictus scabiosae, Lasioglossum politum*, and *Colletes cunicularius*. *Sphecodes ephippius*, *Sphecodes monilicornis*, and *Sphecodes puncticeps* were the most abundant parasitic bees. In addition to ground-nesting bees, several aboveground-nesting bee species were recorded that collected soil as nest-building material. A detailed list of the identified bee species found on nesting hills and their Red-List status is given in Table S2. On nesting hills, both species richness and the abundance of ground-nesting bees increased from the first (2019) to the second (2020) year (Figure 2).

### 3.1. Comparison of the Nesting to the Local Bee Community

**Figure 3:**
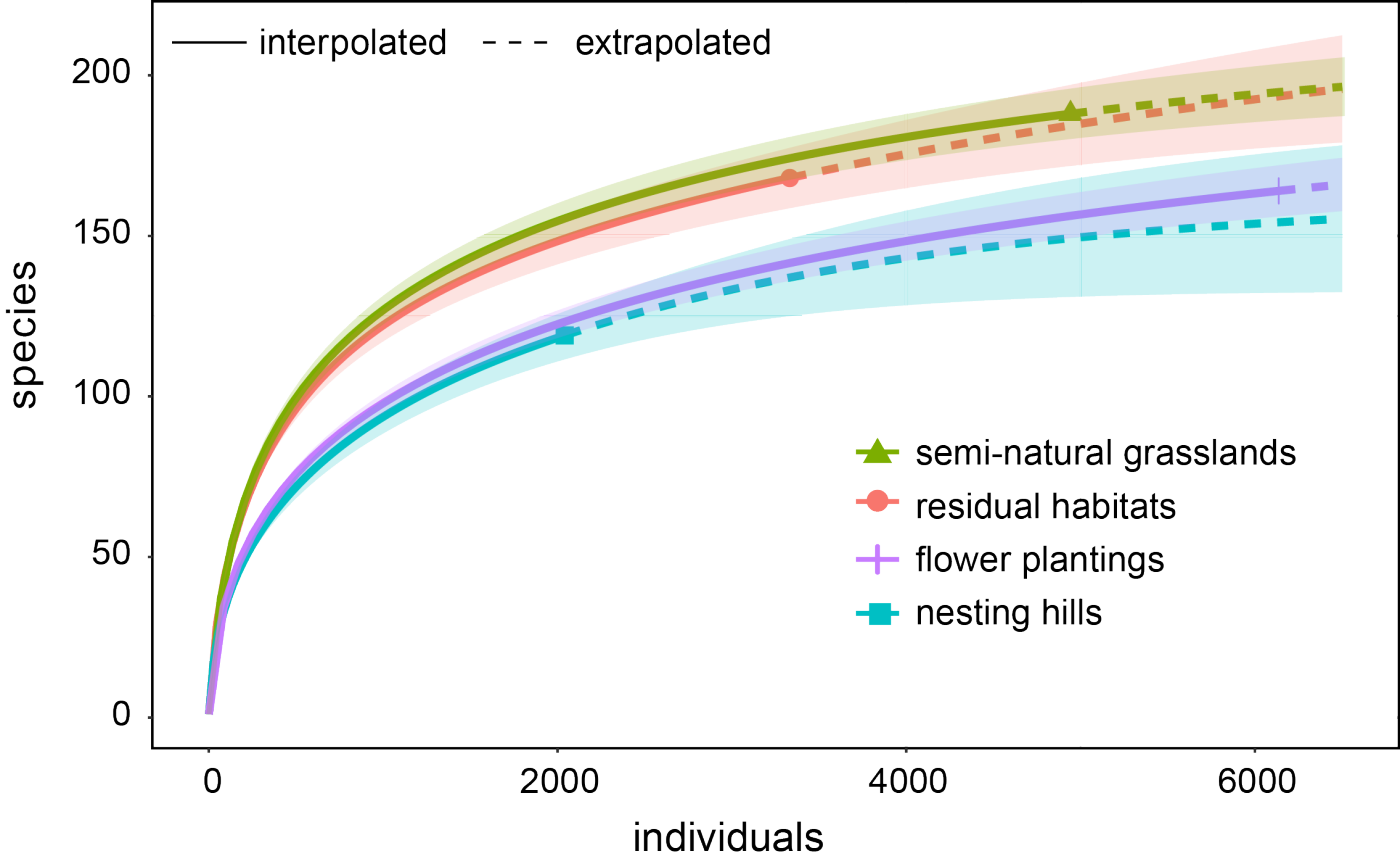
Individual-based randomized species accumulation curves comparing wild bee richness on nesting hills with the three reference habitat types: flower plantings, semi-natural grasslands, and residual habitats. Shaded areas represent 95% confidence intervals.

In total 14,395 ground-nesting bees of 220 species were recorded in the semi-natural grasslands, residual habitats, and flower plantings. The highest numbers of bee species were recorded in semi-natural grasslands and residual habitats, and the highest numbers of individuals were observed at the flower plantings. The number of species recorded at the artificial habitats, nesting hills, and flower plantings reached 72% of the other habitat types (Figure 3). The species accumulation separated for the first and the second year (Figure S1) showed a strong increase in bee species and individuals from the first to the second year only for the nesting hills. The number of bee species did not increase or only slightly increased for the other habitat types or was not different from or decreased for individual numbers. Although species composition on nesting hills differed from that on the three reference habitats, the observed dissimilarities were relatively low and equally pronounced (Table 1).

### 3.2. Abiotic Parameters (Substrate, Soil Temperature, Aspect)

**Figure 4:**
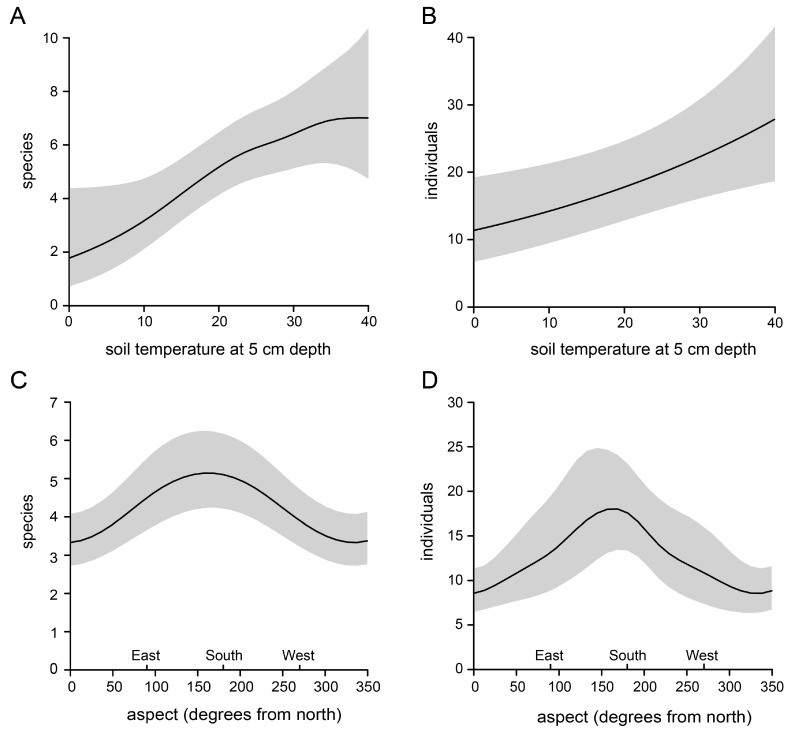
Effect of the abiotic variables (**A**,**B**) soil temperature at a 5 cm depth and (**C**,**D**) aspect (compass direction) on the number of registered bee species and individuals. Predicted values with 95% confidence intervals. Estimated values of the underlying GAMs are provided in the Supplementary Material (Table S4).

Soil temperature at 5 a cm depth of the nesting hills varied between 8 °C (April) and 40.2 °C (August) with a median of 22.5 °C. At a 15 cm depth, soil temperature was lower and varied between 6.7 and 35.5 °C with a median of 19.9 °C. Species richness and bee abundance showed a positive linear relationship to soil temperature at a 5 cm depth (Figure 4a,b, Table S4). The positive effect of soil temperature for species richness flattened out at temperatures higher than 30 °C (Figure 4a). The aspects showed a strong non-linear effect on bee species richness and abundance with a peak around 180°, which represented an aspect facing south (Figure 4c,d, Table S4).

The AIC model selection of the GAMs testing effects on wild bee richness and abundance excluded all tested substrate parameters from the modeling process. Hence, substrate parameters were identified as unimportant predictors for species richness and abundance.

With regard to the effects on the species composition, only the clay content of the soil explained 2.7% (F = 1.5435, *p* = 0.026) of the compositional variation in the dataset. All other substrate parameters were excluded during the automatic model selection of the redundancy analysis.

### 3.3. Landscape Composition (Cover of Semi-Natural Landscape Elements)

**Figure 5:**
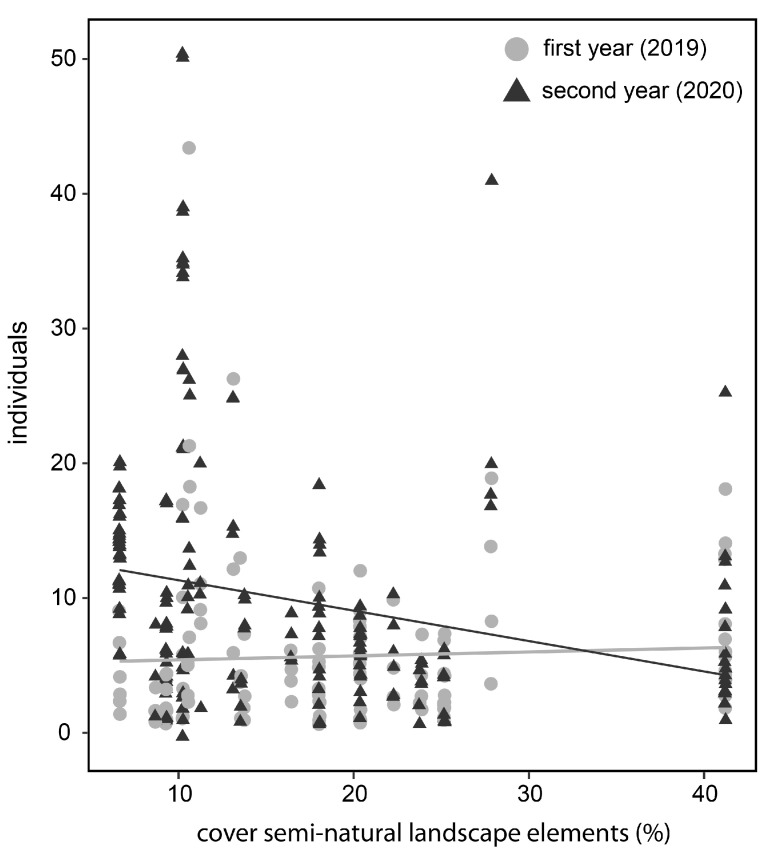
Influence of landscape composition (semi-natural landscape elements) on the number of individuals registered on the nesting hills. The numbers and linear trends (line) are shown for the first (light gray) and the second (dark gray) year after establishment of the nesting hills. Coefficients of the GAM model are given in the Supplementary Material (Table S4).

Bee abundance was significantly influenced by the landscape composition in the second year after establishment of the nesting hills, but not in the first year. The number of bees recorded on the nesting hills increased in research areas with a low cover of semi-natural landscape elements in the surroundings (Figure 5, Table S4).

### 3.4. Pollen Foraging Behaviour

**Figure 6:**
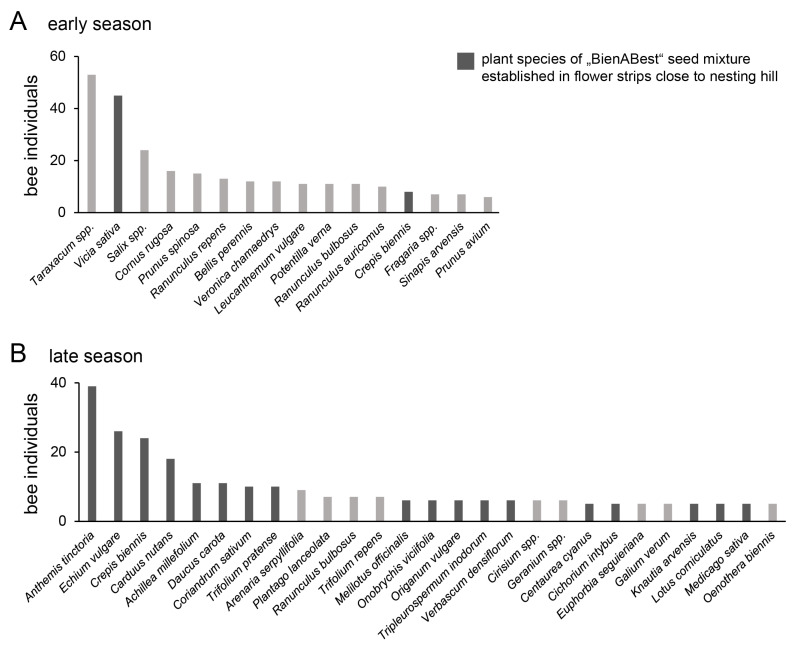
Pollen sources of bee individuals that nested on the nesting hills and carried pollen of a respective plant species, registered in (**A**) April and May (early season) or (**B**) June and July (late season). Plants species that were part of the BienABest seed mixture established in flower plantings close to the nesting hills are shown in dark gray. Only plant species whose pollen was found on at least five bees are included. Plant species are sorted by the total number of samples.

In spring, nesting bees predominately foraged pollen from plants of existing habitats in the surrounding, such as *Taraxacum* spp. or woody plants such as *Salix* spp. (Figure 6A). During the summer, most of the pollen was, in contrast, foraged from the established flower plantings, whereby *Anthemis tinctoria*, *Crepis biennis*, and *Echium vulgare* were particularly attractive (Figure 6B).

## 4. Discussion

Nesting hills can successfully promote wild bees in agricultural landscapes. Even after only two years, we found a species-rich wild bee community with 119 species (37 threatened or rare species according to the Red List) on the nesting hills. The artificial nesting structures attracted approximately 75% of the bees that occurred in the surroundings. The hills were colonized in succession, and nesting hills that were surrounded by a low cover of semi-natural landscape elements showed a particularly strong increase in bee abundance in the second year. Although only the clay content from all substrate parameters had a weak effect on the bee community, the soil temperature strongly affected the species richness and abundance of the bees. Artificial flower plantings in the surrounding showed a similar bee richness compared with the nesting hills and were the source of the majority of pollen that the nesting species collected in the summer months.

### 4.1. Successive Colonization

Our results showed that wild bees colonized the nesting hills successively. The nest colonies of the first year thus acted as a start population in the second year and thereby enhanced the abundance of bees [39,40,41]. For example, *Halictus rubicundus* females overwinter away from the nests from which they originate but return to build new nests close, usually within 50 cm, to the location of their natal nests [42]. As wild bee populations in habitats of the close surroundings in our project did not strongly vary between the years (Figure S1 and [28]), we cannot explain the increase in bees from the first to the second years on the basis of general population shifts. However, our study design cannot completely exclude that population trends were influenced by climatic conditions.

In contrast to our results, nesting structures for ground-nesting bees in another study were densely populated even by the first year, and no further degree of colonization was observed in the second year [15]. However, the soil squares that were provided as nesting structures were only 1 m^2^ in area, whereas our nesting hills had a surface many times larger than that (about 25 m^2^) and, therefore, provided more potential nesting ground. As nesting hills represent a large nesting ground, full colonization takes longer than one season, and therefore, colonization of the nesting hills was probably not completed within the first year.

In our study, bee abundance in landscapes with a low cover of semi-natural landscape elements showed particularly high increases from the first to the second year. Although not yet studied explicitly for agricultural landscapes, studies in highly degraded urban areas suggest that ground-nesting bees suffer from a lack of nesting resources [12,13,43,44]. A high proportion of agriculturally managed and sealed surfaces might have had a similar effect in our landscapes and made the newly offered nesting hills a particularly valuable and therefore attractive resource. On the other hand, a high cover of steep calcareous grassland in some of our research areas probably provided ample nesting ground. Nevertheless, a network of small patches of suitable nesting sites can host a high diversity of bees, as has been shown for wetlands that form spontaneously in arable fields [45]. In our study, we recorded approximately 75% of the ground-nesting bee species that occurred in the surroundings. Consequently, establishing artificial nesting sites are probably an important measure in bee conservation.

### 4.2. Nesting Substrate

Parameters describing the substrate of the nesting hills (pH value, sand, silt content, organic matter) were not correlated with bee richness or abundance. These findings are in line with another study that has also found no correlation between species richness and abundance with soil composition [15]. With regard to bee composition, only the clay content of the substrate shows a weak effect. Whereas high sand content results in a porous substrate, a high proportion of clay results in more compacted soils that are harder to dig through and that hamper the diffusion of oxygen and humidity [46,47]. Furthermore, soil texture influences the stability of the excavated tunnels. Nevertheless, most bee species that have been studied for their soil preferences appear to accept various substrates [17,48]. The substrate of the nesting hills in this study was always taken from the same region and was similar to the soil present in the research areas. This means that the local bee communities were probably adapted to the offered soil types. Their reaction to a substrate that strongly differed from that locally available remains unknown. The provision of various substrates in the same research area in order to offer choice might reveal further valuable information concerning the substrate preferences of wild bees.

### 4.3. Temperature of Nesting Site

A warm nesting substrate positively affects bee size and development rate and therefore is a key factor in the nest-site selection of bees [13]. Accordingly, we found that bee species richness and abundance are highest at sun-exposed sampling plots (southern aspect) with a high soil temperature. For the social species *Lasioglossum malachurum*, for example, warmer nest temperatures were shown to decrease the development time of the offspring of the bees, resulting in higher total colony growth over the season [23]. Our model selection process favors a temperature at a 5 cm depth probably because the nest entrance temperature was identified as a key determinant for foraging activity of bees [25,49]. Females with warmer nest entrances were shown to begin foraging up to three hours earlier than conspecifics nesting in nearby cooler microhabitats [25]. Since most flowering plants offer the highest pollen loads when they open their flowers early in the day [50], a warm surface temperature of the soil represents a major advantage.

At two sampling events, the soil temperature at a 5 cm depth reached particularly high temperature maxima of 40 °C, but the highest temperature measured at a 15 cm depth was about 5 °C lower than that at 5 cm depth. As the brood area of most ground-nesting bees is estimated to be deeper than 10 cm [13,22,51], temperature peaks caused by a hot air temperature and solar radiation are less pronounced [52,53]. Temperatures around 45 °C are predicted to cause serious stress [22]. We observed already a stagnation in the number of active bees at temperatures around 35 °C.

Naturally, soil temperature is directly driven by solar radiation on the nesting patch [20]. For example, nest densities of *Halictus rubicundus* were particularly high at slopes that faced south and that were characterized by high sun exposure [20]. Bees that search for a nesting site at the hills can choose between various slopes and aspects to build their nests, thereby possibly helping to adjust the nest temperature [13,19]. However, ongoing climate change may cause unfavorable nest temperatures, particularly for bees that construct shallow below-ground nests [13,22]. Hill-shaped structures with a slope that catches the sun at a high angle not only facilitate the warming of the soil, but also provide less sun-exposed and cooler microhabitats.

### 4.4. Local bee Communities and Foraging Habitats

Nesting hills apparently attracted wild bees from various habitats in the surroundings because the nesting hills shared species with all of the three reference habitats of semi-natural grasslands, flower plantings, and residual habitats. Pollen analyses revealed that in spring, bees primarily collected pollen from *Taraxacum* spp. and other species that were common in the surrounding habitats. Among them were also woody plants that provide a large amount of nectar and pollen in the spring and are the main feeding source for many bees during this time of the year [54,55]. The floral resources in the nearby established flower plantings were comparably sparse in spring but reached a maximum around mid-summer [28]. In concordance, the adjacent flower plantings acted as the primary source of pollen for the nesting bees in summer. They might have complemented other missing feeding habitats such as flower-rich meadows or ruderal areas [54,55].

## 5. Conclusions

Artificial nesting structures can act as a valuable nesting resource for a broad spectrum of bee species. The nesting hills of our study were particularly frequently colonized in landscapes that were poor in semi-natural habitats that otherwise offered various nesting resources. Nesting hills should be established in a manner that they can persist for several years, because we have shown that they are successively colonized. As high soil temperatures are preferred during nest-site selection, we recommend situating nesting hills on sun-exposed sites that allow a high thermal gain of the substrate. We also recommend the use of a local substrate to match adaptations and needs of the local bee community. Flower plantings in close proximity to the nesting hills provide an important pollen source especially during the summer, but not in spring. We conclude that the provision of a combination of flower-rich habitats and artificial nesting structures represents an effective measure for promoting wild bees, particularly in degraded landscapes.

## Figures and Tables

**Table 1:** Coefficients of the paired PERMANOVA comparing species composition on nesting hills with the three reference habitats.

Pairwise PERMANOVA	df	F	R^2^	*p*
Residual habitat vs. hill	1	3.717	0.023	<0.001
Semi-natural grassland vs. hill	1	3.767	0.023	<0.001
Flower planting vs. hill	1	4.549	0.027	<0.001

## Data Availability

The raw data supporting the conclusions of this article will be made available by the authors, without undue reservation.

## Supplementary Materials

The following supporting information can be downloaded at https://www.mdpi.com/article/10.3390/insects13080726/s1, Figure S1: Individual-based randomized species accumulation curves comparing wild bee richness on nesting hills to the three reference habitat types separated for the first and second year of the study. Table S1: Research areas of the bee survey. Table S2: Identified bee species and number of individuals monitored on the nesting hills. Table S3: Coefficients of the post-hoc comparison of species richness and abundance between the first and the second year. Table S4: Coefficients for models testing the number of species and number of individuals against the explanatory variables. File S1: Construction and management guideline to establish artificial nesting hills for wild bees.
