# Supplementary material for: Artificial Nesting Hills Promote Wild Bees in Agricultural Landscapes
Source: Insects. 2022 Aug 14;13(8):726. doi: 10.3390/insects13080726 (PMC9409424; doi:10.3390/insects13080726)
Supplement: Supplementary file 1 [file insects-13-00726-s001.zip › File S1.pdf]

File S1

Neumüller, Burger et al. (2022) Artificial nesting hills promote wild bees in agricultural landscapes. *Insects*

# Construction of an artificial nesting hill for wild bees

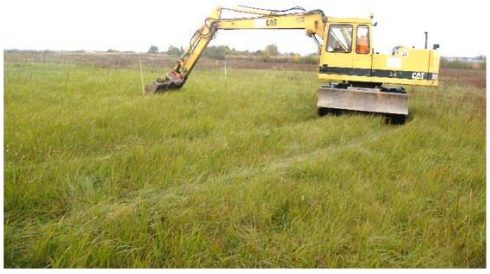

Establishment of the construction area at a sun-exposed site where the nesting hill can exist for several years.

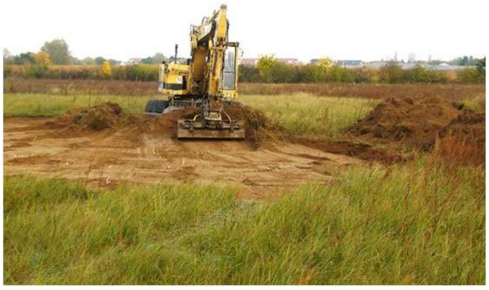

Removing of the surface soil layer beneath or even around the hill to get off plants and most of their roots.

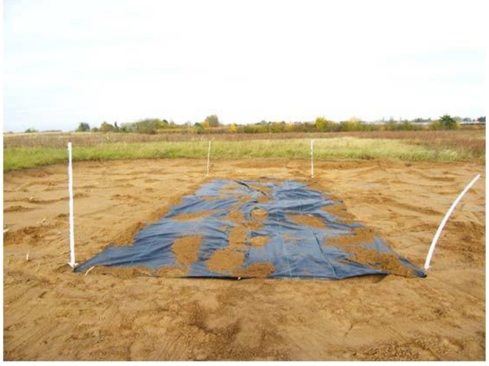

A weed foil can be used to prevent plants from growing through the nesting hill, not obligatory used.

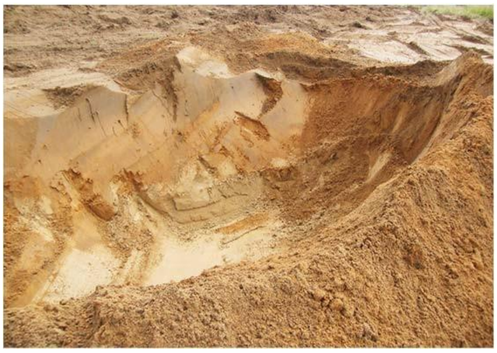

Subsoil is used as substrate (approx. 40 m3), usually from construction areas of the region to minimize transport costs and to match local nesting grounds. The low seedbank of the subsoil prevents excessive plant growth. Compact substrate can be mixed with a third of sand.

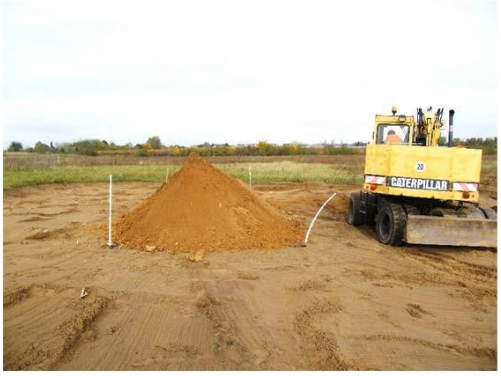


Construction of the nesting hills using a bagger.

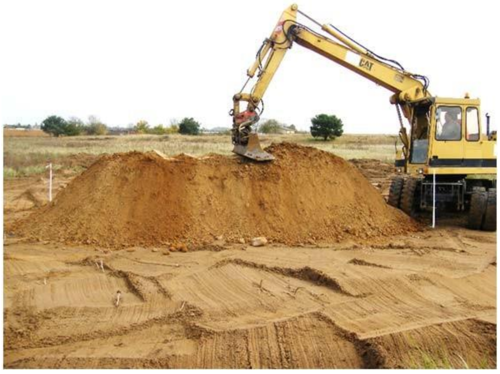

Compressing of the substrate as an important intermediate step.

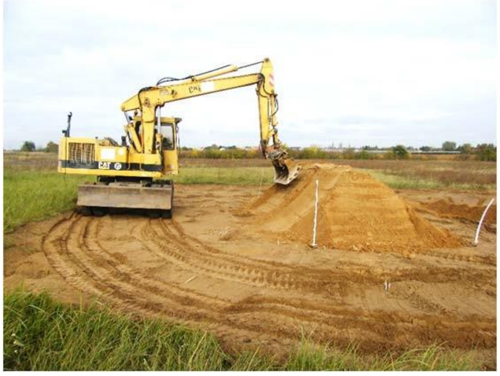

Compressing of the substrate to modulate form and size.

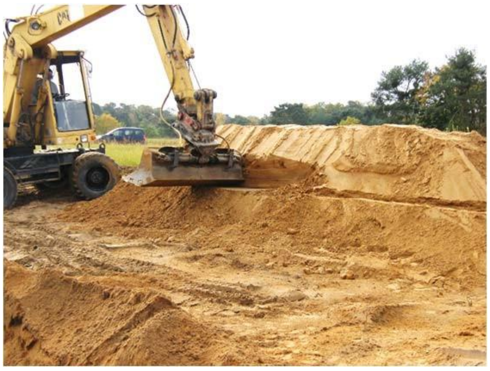

Horizontal step at a height of 1m to facilitate access for management tasks.

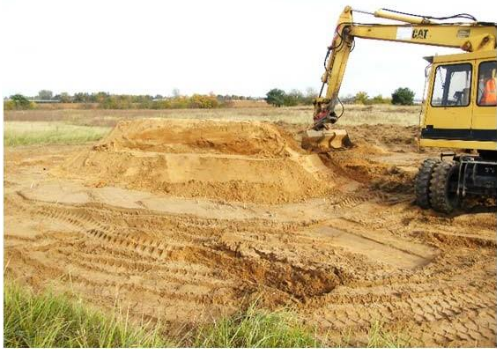

Modulation of the nesting hill.

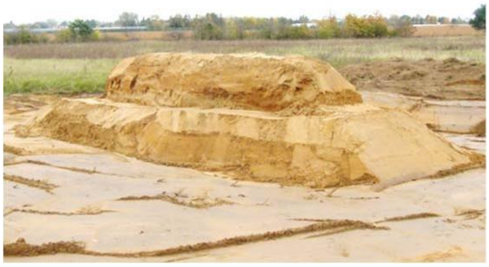

Final nesting hill with a size of 3m width, 9m length, 1.6m height.

photos S. Krausch

# Management of artificial nesting hills for wild bees

Despite measures to prevent plant growth on the nesting hills, spontaneous growth of plants occurred on all nesting hills. Plants with roots were dug out (e.g. digging tools for docks/*Rumex* sp.) or scorched off (flame gun) each autumn/winter after bee activity on nesting hills had ceased. Single plants were also removed or cut (e.g. brushcutter) before seeds were ripe once or twice a year during the bee season.

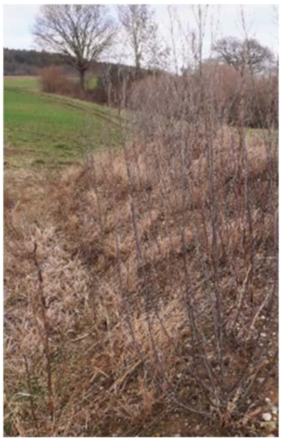
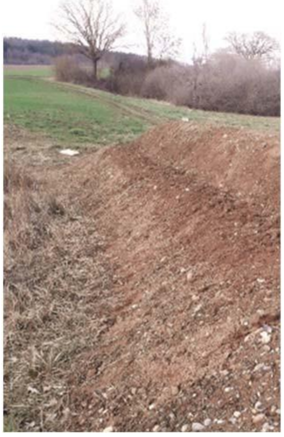

Nesting hill before (left) and after (right) management activities in the autumn (photos W. Matt).

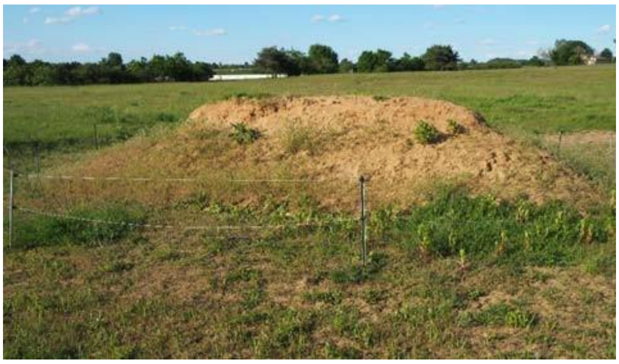

Regularly managed nesting hill (same hill as shown in the construction guide). Single plants or other structures on the hills can promote nesting activity by bees but were at least partly removed to prevent excessive plant growth. A fence can be necessary to prevent the hill from trampling down e.g. by sheeps (photo A. Mayr).

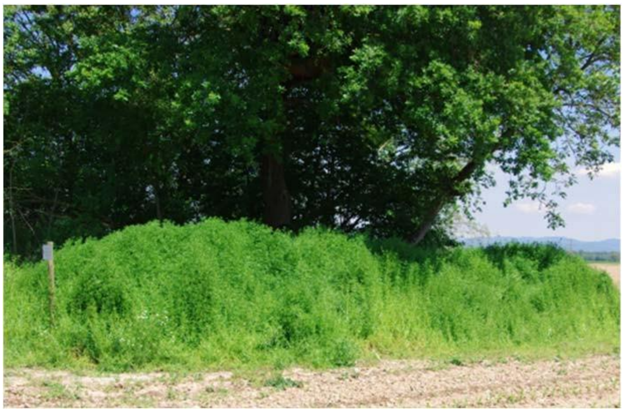
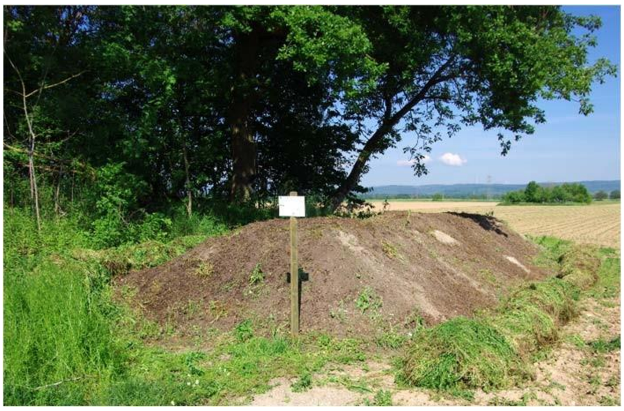

Example of a nesting hill that was built with surface soil with high organic content and seedbank, which led to excessive plant growth (left) and afforded high management efforts to maintain open soil areas (right). The shade of the trees can also negatively influence the nesting activities by bees. The sign informs the public about the procedure to conserve wild bees (photo S. Hopfenmüller).
