# Supplementary material for: Artificial Nesting Hills Promote Wild Bees in Agricultural Landscapes
Source: Insects. 2022 Aug 14;13(8):726. doi: 10.3390/insects13080726 (PMC9409424; doi:10.3390/insects13080726)
Supplement: Supplementary file 1 [file insects-13-00726-s001.zip › SupplementaryMaterial.pdf]

This document provides electronic supplementary material for the article titled:

Artificial nesting hills promote wild bees in agricultural landscapes

Ulrich Neumüller, Hannah Burger, Antonia Veronika Mayr, Sebastian Hopfenmüller, Nadine Herwig, Ronald Burger, Olaf Diestelhorst, Katrin Emmerich, Mare Haider, Manuel Kiefer, Jonas Konicek, Johann-Christoph Kornmilch, Sabrina Krausch, Marina Moser, Christoph Saure, Arno Schanowski, Erwin Scheuchl, Julia Sing, Max Wagner, Julia Witter, Hans Richard Schwenninger, Manfred Ayasse

Content

Table S1 - Research areas of the bee survey

Table S2 - Identified bee species and number of individuals monitored on the nesting hills.

Table S3 - Coefficients of the post-hoc comparison of species richness and abundance between the first and the second year

Figure S1 - Individual-based randomized species accumulation curves comparing wild bee richness on nesting hills to the three reference habitat types separated for the first and second year of the study

Table S4 - Coefficients for models testing the number of species and number of individuals against the explanatory variables

See Appendix A for a construction and management guideline for establishing artificial nesting hills for wild bees

Table S1. Research areas of the bee survey. Areas where the extended monitoring (4 instead of 2 monitoring events per year and pollen survey) took place are marked with an asterisk.

Research area	Coordinates
Reichenbach*	N48.62 E9.73
Eselsburger Tal*	N48.60 E10.18
Hirschtal*	N48.69 E10.03
Obergailingen	N47.69 E8.77
Kappel-Grafenhausen	N48.29 E7.73
Hügelsheim	N48.79 E8.091
Elsbachtal	N51.08 E6.52
Rekultivierung Garzweiler	N51.02 E6.54
Annweiler	N49.21 E7.97
Rohrbach	N49.13 E8.11
Golßen	N51.98 E13.53
Temmen	N53.10 E13.77
Frankenbacher Schotter*	N49.14 E9.16
Dettenheim*	N49.17 E8.38
Griesheimer Düne*	N49.84 E8.56
Rotböll*	N49.92 E8.62
Landshut	N48.44 E12.11
Dingolfing-Landau	N48.68 E12.55
Guest	N54.04 E13.44
Wangelkow	N53.91 E13.81

Table S2. Identified bee species and number of individuals monitored on the nesting hills. The Red list status is categorized according to Westrich et al. (2011). Statistical analyses were performed on ground-nesting bees.

Species	Individuals	Red list	Nesting
Andrena agilissima (Scopoli, 1770)	1	3	en
Andrena alfkenella Perkins, 1914	1	LC	en
Andrena barbilabris (Kirby, 1802)	12	LC	en
Andrena bicolor Fabricius, 1775	1	*	en
Andrena carantonica Pérez, 1902	3	*	en
Andrena cineraria (Linnaeus, 1758)	7	*	en
Andrena curvungula Thomson, 1870	1	3	en
Andrena dorsata (Kirby, 1802)	5	*	en
Andrena flavipes Panzer, 1799	130	*	en
Andrena florivaga Eversmann, 1852	1	*	en
Andrena haemorrhoa (Fabricius, 1781)	3	*	en
Andrena hattorfiana (Fabricius, 1775)	1	3	en
Andrena labiata Fabricius, 1781	1	*	en
Andrena minutula (Kirby, 1802)	42	*	en
Andrena minutuloides Perkins, 1914	7	*	en
Andrena nigriceps (Kirby, 1802)	2	2	en
Andrena nigroaenea (Kirby, 1802)	1	*	en
Andrena nigrospina Thomson, 1872	3	3[1]	en
Andrena nitida (Müller, 1776)	2	*	en
Andrena niveata Friese, 1887	2	3	en
Andrena ovatula (Kirby, 1802)	6	*	en
Andrena pilipes Fabricius, 1781	1	3	en
Andrena propinqua Schenck, 1853	1	*[2]	en
Andrena rhenana Stöckhert, 1930	1	NT	en
Andrena subopaca Nylander, 1848	4	*	en
Andrena vaga Panzer, 1799	1	*	en
Andrena viridescens Viereck, 1916	6	LC	en
Trachusa byssina (Panzer, 1798)	1	3	en
Anthidium manicatum (Linnaeus, 1758)	1	*	hy
Anthidium oblongatum (Illiger, 1806)	3	LC	hy
Anthidium punctatum Latreille, 1809	2	LC	en
Anthidiellum strigatum (Panzer, 1805)	1	LC	hy
Anthophora bimaculata (Panzer, 1798)	7	3	en

Anthophora furcata (Panzer, 1798)	1	LC	hy
Anthophora plumipes (Pallas, 1772)	3	*	en
Anthophora retusa (Linnaeus, 1758)	1	LC	en
Bombus humilis Illiger, 1806	3	3	hy
Bombus lapidarius (Linnaeus, 1758)	13	*	en
Bombus pascuorum (Scopoli, 1763)	2	*	en
Bombus sylvarum (Linnaeus, 1761)	2	LC	en
Bombus terrestris (Linnaeus, 1758)	50	*	en
Ceratina chalybea Chevrier, 1872	1	3	hy
Ceratina cucurbitina (Rossi, 1792)	2	*	hy
Ceratina cyanea (Kirby, 1802)	2	*	hy
Chelostoma florisomne (Linnaeus, 1758)	74	*	hy
Coelioxys afra Lepeletier, 1841	19	3	en (pa)
Coelioxys conica (Linnaeus, 1758)	2	LC	en (pa)
Coelioxys conoidea (Illiger, 1806)	2	3	en (pa)
Colletes cunicularius (Linnaeus, 1761)	139	*	en
Colletes fodiens (Geoffroy, 1785)	9	3	en
Colletes similis Schenck, 1853	1	LC	en
Dasypoda hirtipes (Fabricius, 1793)	1	LC	en
Epeoloides coecutiens (Fabricius, 1775)	1	*	en (pa)
Epeolus variegatus (Linnaeus, 1758)	7	LC	en (pa)
Eucera nigrescens Pérez, 1879	2	*	en
Halictus leucaheneus Ebmer, 1972	6	3	en
Halictus maculatus Smith, 1848	10	*	en
Halictus quadricinctus (Fabricius, 1776)	124	3	en
Halictus scabiosae (Rossi, 1790)	187	*	en
Halictus sexcinctus (Fabricius, 1775)	99	3	en
Halictus simplex Blüthgen, 1923	32	*	en
Halictus subauratus (Rossi, 1792)	77	*	en
Halictus tumulorum (Linnaeus, 1758)	14	*	en
Heriades truncorum (Linnaeus, 1758)	2	*	hy
Hoplitis adunca (Panzer, 1798)	28	*	hy
Hoplitis leucomelana (Kirby, 1802)	2	*	hy
Hoplitis papaveris (Latreille, 1799)	3	1	en
Hylaeus angustatus (Schenck, 1861)	1	*	hy
Hylaeus brevicornis Nylander, 1852	1	*	hy
Hylaeus communis Nylander, 1852	5	*	hy

Hylaeus kahri Förster, 1871	1	*	hy
Hylaeus lineolatus (Schenck, 1861)	2	NE	hy
Hylaeus variegatus (Fabricius, 1798)	4	LC	en
Lasioglossum aeratum (Kirby, 1802)	3	3	en
Lasioglossum albipes (Fabricius, 1781)	2	*	en
Lasioglossum brevicorne (Schenck, 1868)	2	3	en
Lasioglossum calceatum (Scopoli, 1763)	9	*	en
Lasioglossum clypeare (Schenck, 1853)	1	2	en
Lasioglossum costulatum (Kriechbaumer, 1873)	1	3	en
Lasioglossum fulvicorne (Kirby, 1802)	5	*	en
Lasioglossum glabriusculum (Morawitz, 1872)	46	*	en
Lasioglossum intermedium (Schenck, 1868)	1	3	en
Lasioglossum interruptum (Panzer, 1798)	17	3	en
Lasioglossum laticeps (Schenck, 1868)	34	*	en
Lasioglossum lativentre (Schenck, 1853)	4	LC	en
Lasioglossum leucozonium (Schrank, 1781)	8	*	en
Lasioglossum lucidulum (Schenck, 1861)	21	*	en
Lasioglossum malachurum (Kirby, 1802)	46	*	en
Lasioglossum minutissimum (Kirby, 1802)	3	*	en
Lasioglossum morio (Fabricius, 1793)	48	*	en
Lasioglossum nitidiusculum (Kirby, 1802)	1	LC	en
Lasioglossum nitidulum (Fabricius, 1804)	1	*	en
Lasioglossum pauxillum (Schenck, 1853)	131	*	en
Lasioglossum politum (Schenck, 1853)	147	*	en
Lasioglossum punctatissimum (Schenck, 1853)	2	*	en
Lasioglossum pygmaeum (Schenck, 1853)	1	NE	en
Lasioglossum quadrinotatulum (Schenck, 1861)	3	3	en
Lasioglossum semilucens (Alfken, 1914)	1	*	en
Lasioglossum sexnotatum (Kirby, 1802)	1	3	en
Lasioglossum subhirtum (Lepeletier, 1841)	1	3	en
Lasioglossum villosulum (Kirby, 1802)	131	*	en
Lasioglossum zonulum (Smith, 1848)	3	*	en
Megachile circumcincta (Kirby, 1802)	2	LC	hy
Megachile ericetorum Lepeletier, 1841	2	*	en/hy
Megachile pilidens Alfken, 1924	18	3	en
Melitta leporina (Panzer, 1799)	1	*	en
Nomada alboguttata Herrich-Schäffer, 1839	1	*	en (pa)

Nomada argentata Herrich-Schäffer, 1839	3	2	en (pa)
Nomada atroscutellaris Strand, 1921	4	LC	en (pa)
Nomada bifasciata Olivier, 1811	1	*	en (pa)
Nomada distinguenda Morawitz, 1873	2	NE	en (pa)
Nomada fabriciana (Linnaeus, 1767)	2	*	en (pa)
Nomada flavoguttata (Kirby, 1802)	12	*	en (pa)
Nomada flavopicta (Kirby, 1802)	1	*	en (pa)
Nomada fucata Panzer, 1798	13	*	en (pa)
Nomada fulvicornis Fabricius, 1793	1	*	en (pa)
Nomada lathburiana (Kirby, 1802)	2	*	en (pa)
Nomada panzeri Lepeletier, 1841	2	*	en (pa)
Nomada sheppardana (Kirby 1802)	2	*	en (pa)
Nomada succincta Panzer, 1798	1	*	en (pa)
Nomioides minutissimus (Rossi, 1790)	3	2	en
Osmia bicolor (Schrank, 1781)	1	*	hy (helico)
Osmia aurulenta (Panzer, 1799)	1	*	hy (helico)
Osmia bicornis (Linnaeus, 1758)	19	*	hy
Osmia brevicornis (Fabricius, 1798)	2	NE	hy
Osmia caerulescens (Linnaeus, 1758)	2	*	hy
Osmia spinulosa (Kirby, 1802)	1	3	hy (helico)
Panurgus calcaratus (Scopoli, 1763)	4	*	en
Sphecodes albilabris (Fabricius, 1793)	27	*	en (pa)
Sphecodes crassus Thomson, 1870	16	*	en (pa)
Sphecodes cristatus von Hagens, 1882	5	NE	en (pa)
Sphecodes ephippius (Linnaeus, 1767)	45	*	en (pa)
Sphecodes ferruginatus von Hagens, 1882	3	*	en (pa)
Sphecodes gibbus (Linnaeus, 1758)	29	*	en (pa)
Sphecodes hyalinatus von Hagens, 1882	2	*	en (pa)
Sphecodes longulus von Hagens, 1882	6	*	en (pa)
Sphecodes miniatus von Hagens, 1882	9	*	en (pa)
Sphecodes monilicornis (Kirby, 1802)	35	*	en (pa)
Sphecodes pellucidus Smith, 1845	7	LC	en (pa)
Sphecodes pseudofasciatus Blüthgen, 1925	1	DD	en (pa)
Sphecodes puncticeps Thomson, 1870	35	*	en (pa)
Sphecodes reticulatus Thomson, 1870	2	*	en (pa)
Sphecodes rufiventris (Panzer, 1798)	1	*	en (pa)

Red list categories:
1 = critically endangered (CR)
2 = endangered (EN)
3 = vulnerable (VU)
NT = near threatened
LC = least concern
DD = data deficient
NE = not evaluated
* = not threatened

[1]Red list status unknown, given status of *Andrena pilipes*
[2]Red list status unknown, given status of *Andrena dorsata*

Nesting behaviour:
en = endogeic (nesting in the ground)
en/hy = endogeic/hypergeic (nesting in the ground or close to the ground)
hy = hypergeic (nesting in a variety of structures above ground)
helico = helicophile (nesting in snail shells)
pa = parasitic

Table S3. Coefficients of the post-hoc comparison of species richness and abundance between the first and the second year. Significant values ($p < 0.05$) are printed in bold.

Species richness					
contrast	*estimate*	*SE*	*df*	*t*	*p-value*
2019 - 2020	-0.209	0.0638	278	-3.269	**0.001**
Abundance					
contrast	*estimate*	*SE*	*df*	*t*	*p-value*
2019 - 2020	-0.435	0.0836	275	-5.204	**<0.001**

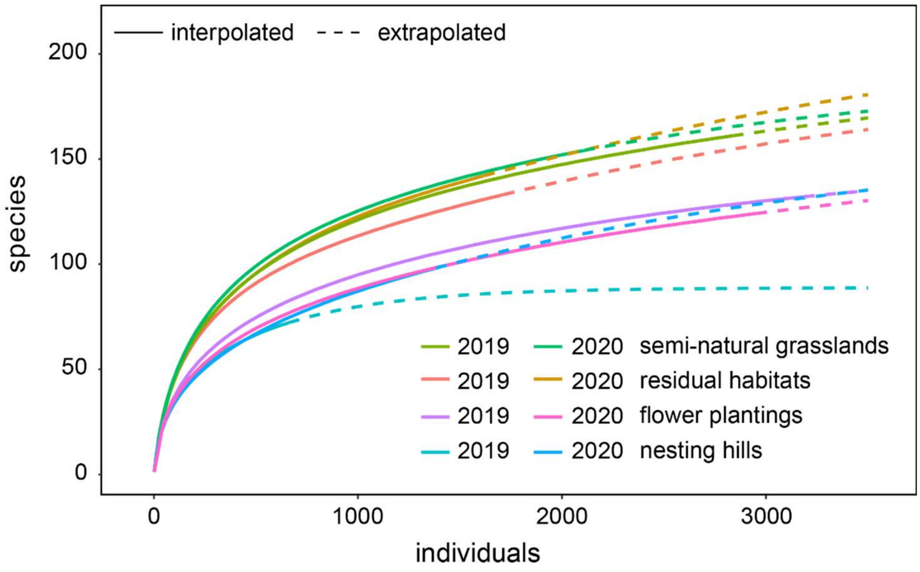

Figure S1. Individual-based randomized species accumulation curves comparing wild bee richness on nesting hills to the three reference habitat types flower plantings, semi-natural grasslands, and residual habitats separated for the first and second year of the study.

Table S4. Coefficients for models testing the number of species and number of individuals against the explanatory variables. Significant values ($p < 0.05$) are printed in bold.

Number of species

smooth terms	*Estimated Df*	*Chi.sq*	*p-value*
Aspect (degrees from North)	2.667	39.780	**<0.001**
Soil temperature 5cm	2.517	16.430	**0.001**
Day of year	2.569	19.010	**<0.001**
Hill ID (random variable)	13.611	60.530	**<0.001**
Year (random variable)	0.924	18.990	**<0.001**

Number of individuals

Parametric coefficients	*Estimate*	*Std. Error*	*z*	*p-value*
Year (2019 vs. 2020)	0.823	0.213	3.863	**<0.001**
Semi-natural landscape elements	-0.001	0.019	-0.075	0.940
Year:Semi-natural landscape elements (2019 vs. 2020)	-0.022	0.008	-2.762	**0.006**

smooth terms	*Estimated Df*	*Chi.sq*	*p-value*
Aspect (degrees from North)	3.009	39.660	**<0.001**
Soil temperature 5cm	1.002	6.835	**0.009**
Day of year	3.116	29.271	**<0.001**
Hill ID (random variable)	15.090	154.709	**<0.001**
Year (random variable)	0.000	0.000	**0.008**

Reference

Westrich, P.; Frommer, U.; Mandery, K.; Riemann, H.; Ruhnke, H.; Saure, C.; Voith, J. Rote Liste und Gesamtartenliste der Bienen (Hymenoptera, Apidae) Deutschlands. In *Rote Liste gefährdeter Tiere, Pflanzen und Pilze Deutschlands. Band 3: Wirbellose Tiere (Teil 1)*, Naturschutz, B.f., Ed.; Naturschutz und Biologische Vielfalt: Bonn-Bad Godesberg, 2011; Volume 70, pp. 373-416.
